# Retinal Layer Separation (ReLayS) method enables the molecular analysis of photoreceptor segments and cell bodies, as well as the inner retina

**DOI:** 10.1038/s41598-022-24586-8

**Published:** 2022-11-23

**Authors:** Vyara Todorova, Luca Merolla, Duygu Karademir, Gabriele M. Wögenstein, Julian Behr, Lynn J. A. Ebner, Marijana Samardzija, Christian Grimm

**Affiliations:** Laboratory for Retinal Cell Biology, Department of Ophthalmology, University Hospital Zurich, University of Zurich, 8952 Schlieren, Zurich Switzerland

**Keywords:** Isolation, separation and purification, Retina

## Abstract

Understanding the physiology of the retina, and especially of the highly polarized photoreceptors, is essential not only to broaden our knowledge of the processes required for normal vision, but also to develop effective therapies to prevent or slow retinal degenerative diseases. However, the molecular analysis of photoreceptors is a challenge due to the heterogeneity of the retinal tissue and the lack of easy and reliable methods for cell separation. Here we present the ReLayS method—a simple technique for the separation of photoreceptor segments (PS) containing both inner and outer segments, outer nuclear layer (ONL), and inner retina (InR) that contains the remaining retinal layers. The layer-specific material isolated from a mouse half-retina with the ReLayS method was sufficient for protein isolation and Western blotting or RNA isolation and real-time PCR studies. The separation of PS, ONL, and InR was successfully validated by Western blotting and real-time PCR using proteins and genes with known expression profiles within the retina. Furthermore, the separation of the PS from the ONL enabled the detection of light-driven translocation of transducin from the PS to the soma. ReLayS is a simple and useful method to address protein and possibly metabolites distribution in photoreceptor compartments in various situations including development, ageing, and degenerative diseases.

## Introduction

**Figure 1:**
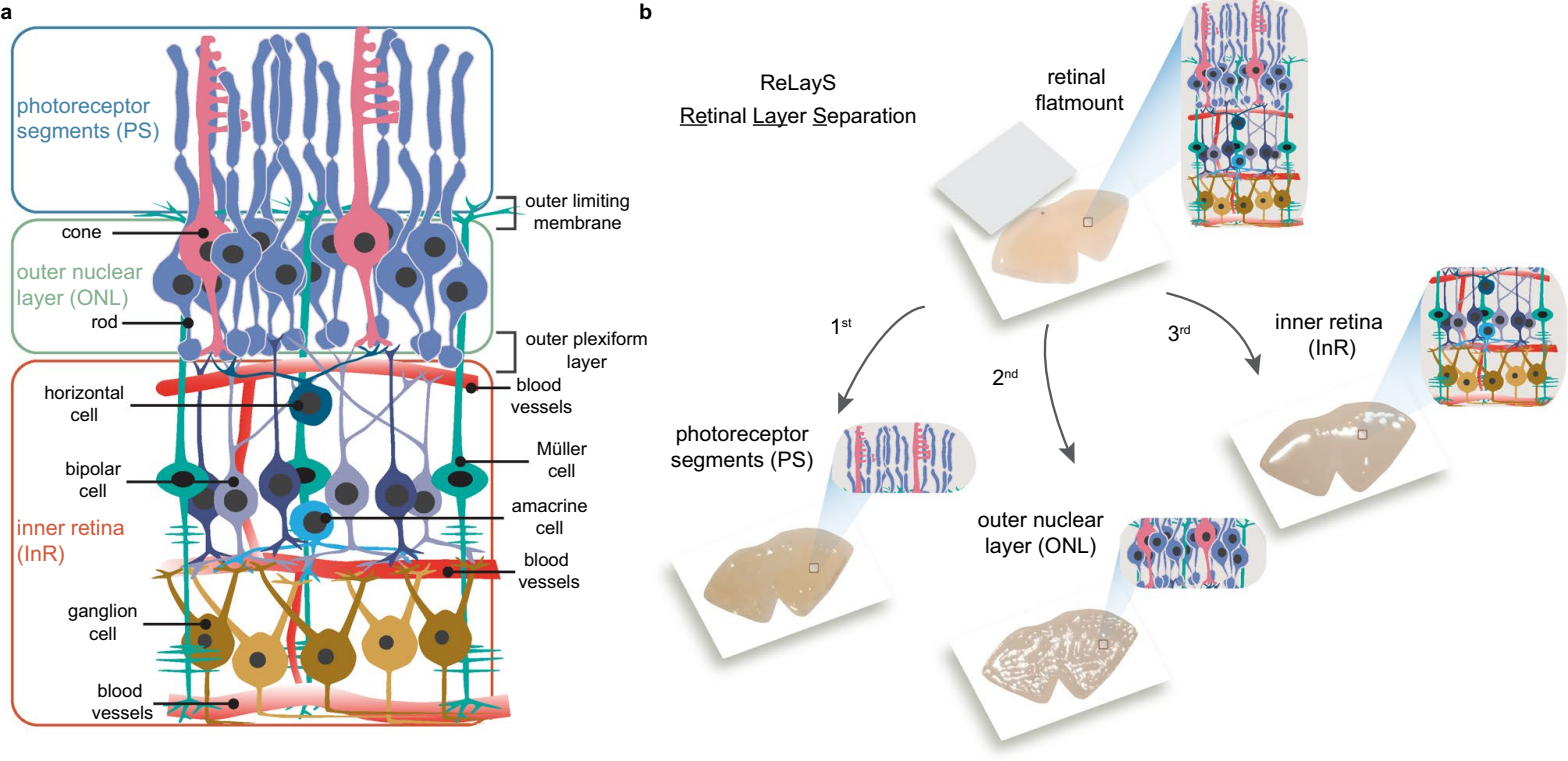
Scheme of the ReLayS method. (**a**) Schematic representation of the retina showing the localization of the seven major cell types in the retina and the two breaking points for ReLayS - the outer limiting membrane and the outer plexiform layer. (**b**) Schematic representation and representative photographs of photoreceptor segments (PS), outer nuclear layer (ONL), and inner retina (InR) as they are separated by ReLayS. Images were created in Adobe Illustrator CS6.

The retina is a highly heterogeneous tissue with seven major cell types arranged in three cellular layers connected by two synaptic layers, providing visual input detection and initial signal processing^1^ (Fig. 1a). The outermost cell layer is populated by rod and cone photoreceptors. Photoreceptors are highly polarized cells consisting of a light-sensitive outer segment (OS), an inner segment (IS) containing the metabolic machinery of the cell, a soma with the nucleus in the outer nuclear layer (ONL), and an axon ending in a synaptic terminal in the outer plexiform layer (OPL). In the OPL, photoreceptors connect to the second-order neurons of the inner retina, which itself is morphologically and functionally organized in several layers^2^. The inner nuclear layer (INL) contains cell bodies of bipolar, horizontal, amacrine, and Müller glial cells. Neurotransmission from second-order neurons in the INL to retinal ganglion cells occurs in the inner plexiform layer (IPL). The ganglion cell bodies populate the ganglion cell layer (GCL) together with some amacrine cells and astrocytes. Microglia localize to the inner retina, but can migrate to the outer retina upon activation by photoreceptor damage or stress.

Retinal pathologies associated with photoreceptor degeneration such as age-related macular degeneration, diabetic retinopathy, and retinitis pigmentosa comprise a large proportion of untreatable blindness globally^3^. To understand the underlying pathological mechanisms and develop successful therapies to preserve vision, it is essential to study the biochemical and molecular events in photoreceptors, ideally on a subcellular level. However, isolation of photoreceptor inner and outer segments as well as of photoreceptor cell bodies for biochemical and molecular analysis is a major challenge. The most widely used method for isolation of photoreceptor outer segments was originally developed for bovine retinas^4^ but has been adapted for other eyes including those of pigs^5^, amphibians^6^, and dogs^7^. In this method, the tissue is mechanically ruptured and the outer segments are purified by a sucrose gradient^4^. However, this method has two major limitations. First, it requires a sizable amount of retinal tissue, and second, inner segments and photoreceptor cell bodies are lost in the procedure. Other methods for the isolation of retinal cells are based on tissue dissociation^8,9^. In most instances, those methods also require pooling of several retinas and involve rather complicated and long-lasting procedures, including manual sorting based on cell morphology or flow cytometry. Laser capture microdissection is an alternative technique for the isolation of small tissue samples and is compatible with mass spectrometry applications as well as DNA and RNA profiling^10,11^. Unfortunately, the limiting amount of the samples as well as the tissue processing makes it incompatible with molecular analysis such as Western blotting and metabolite detection assays. Serial tangential sectioning of flat-mounted frozen retinas is a method well suited for protein analysis using Western blotting^12^. This method depends on the perfect alignment of the retinal layers with the cutting plane of the cryostat knife to collect similar fractions from different retinas. Because the collection of many fractions from a single retina is possible by this method, it is well suited for high resolution localization studies. Highly expressed proteins can be detected by Western blotting, proteins with reduced expression may need more sensitive methods such as mass spectrometry. Two peeling methods were introduced by Rose et al. for the isolation of photoreceptor outer and/or inner segments from the mouse retina^13^. The first method requires lyophilization of the retina and uses Scotch tape for peeling the photoreceptor outer and inner segments. The second method uses Whatman filter paper to separate the photoreceptor outer segments from retinal tissue. Isolation of the ONL is, however, not provided in those methods.

Here we introduce a simple method for retinal layer separation (ReLayS) from mouse eyes that efficiently and reproducibly isolates three fractions that contain the photoreceptor inner and outer segments (PS), the outer nuclear layer (ONL), and the inner retina (InR) with all other layers. Samples prepared with the ReLayS method from individual half-retinas provide sufficient material for Western blotting or real-time PCR, allowing the study of the subcellular molecular composition of photoreceptors. The ReLayS method was validated by Western blotting and quantitative RT-PCR using several representative proteins and genes for photoreceptor compartments and different retinal cell types.

## Results

### Protein and RNA concentrations in isolated retinal layers

**Figure 2:**
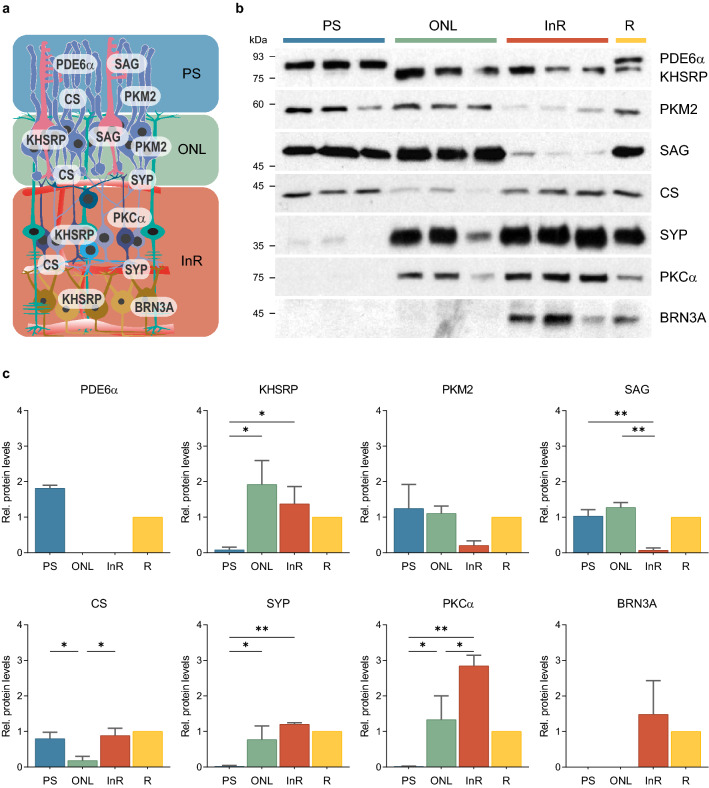
Analysis of protein distribution in ReLayS samples. (**a**) Schematic representation of the retina showing the known distribution of the proteins used in the Western blot analyses. Image was created in Adobe Illustrator CS6. (**b**) The presence of PDE6$\upalpha$, KHSRP, PKM2, SAG, CS, SYP, PKC$\upalpha$, and BRN3A in PS, ONL, InR, and whole retina (R) was analysed by Western blotting. n = 3. (**c**) Quantitative analysis of the signals from the Western blots shown in (**b**). Protein levels in PS (blue), ONL (green), and InR (red) are represented as means $\pm$ SD relative to R (yellow). Statistics: one-way ANOVA with Holm-Sidak’s multiple comparisons test. *p $\le$ 0.05, **p $\le$ 0.01.

**Figure 3:**
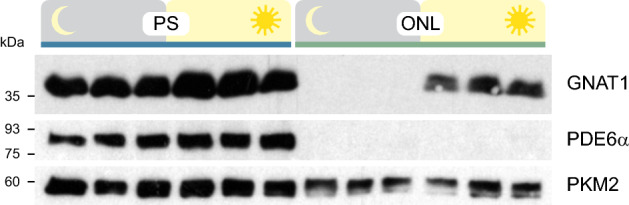
Light-induced translocation of transducin in photoreceptors. Western blot analysis showing the distribution of the $\upalpha$-subunit of rod transducin (GNAT1) in the photoreceptor segments (PS) and outer nuclear layer (ONL) of dark- (left) and light- (right) adapted mice. PDE6$\upalpha$ located exclusively in PS samples was used as a control for layer separation and PKM2 as a loading control.

**Figure 4:**
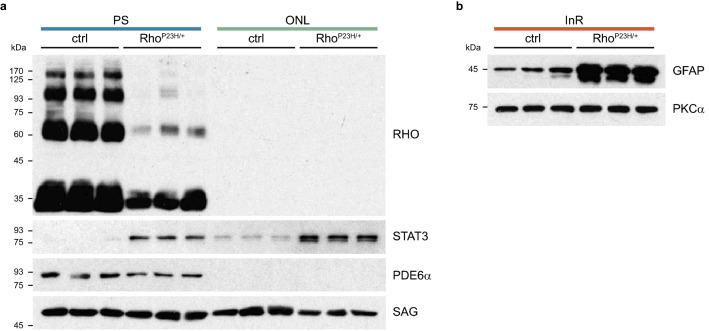
Photoreceptor degeneration and reactive gliosis in the retina of heterozygous P23H knock-in mice. (**a**) The levels of rhodopsin (RHO) and STAT3 were analysed in the PS and ONL of control and 3 months old Rho^P23H/+^ mice. PDE6$\upalpha$ located exclusively in PS samples was used as a control for layer separation and SAG as a loading control. (**b**) The levels of GFAP were analysed in the InR of control and 3 months old Rho^P23H/+^ mice. PKC$\upalpha$ was used as a loading control. n = 3.

**Figure 5:**
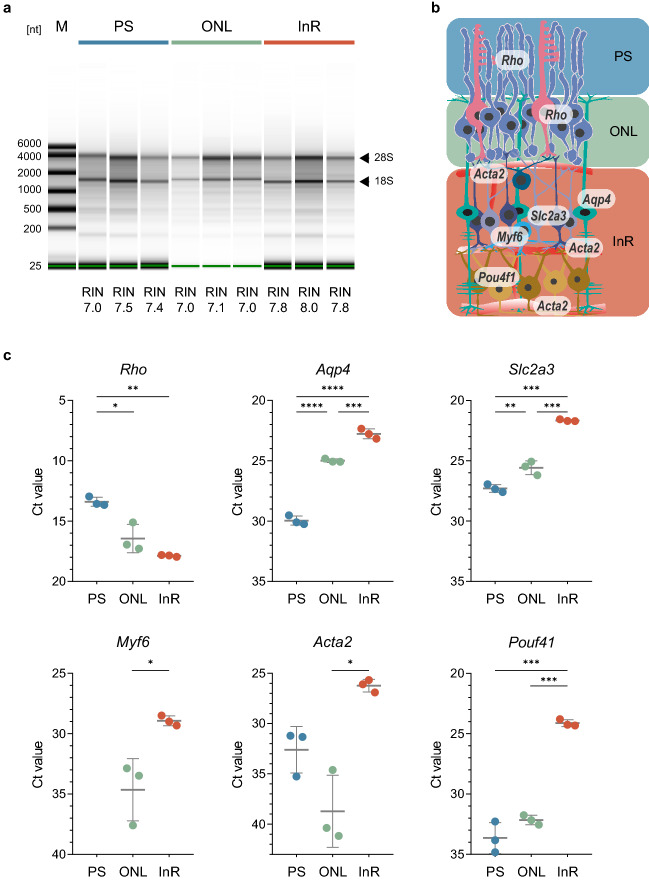
Analysis of RNA expression in ReLayS samples. (**a**) Assessment of RNA integrity by electrophoresis of total RNA from PS, ONL, and InR samples and RNA integrity number (RIN) calculation. (**b**) Schematic representation of the retina showing spatial distribution of the genes analysed by real-time PCR. Image was created in Adobe Illustrator CS6. (**c**) Cycle-threshold (Ct) values of indicated mRNAs in PS, ONL, and InR samples. Shown are individual data points and means $\pm$ SD of n = 3. Statistics: Student’s *t*-test (for *Myf6*) or one-way ANOVA with Holm-Sidak’s multiple comparisons test. *p $\le$ 0.05, **p $\le$ 0.01, ***p $\le$ 0.001, ****p $\le$ 0.0001.

**Figure 6:**
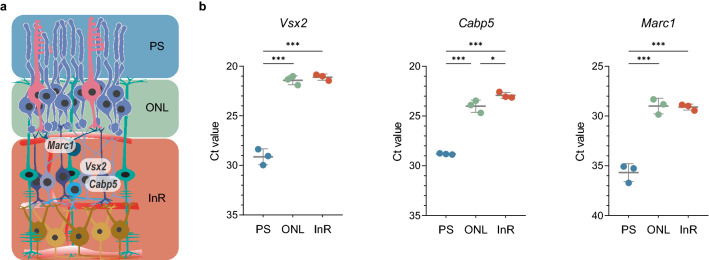
Analysis of bipolar and horizontal cells RNA distribution in ReLayS samples. (**a**) Schematic representation of the retina showing spatial expression of *Vsx2* and *Cabp5* in bipolar and *Marc1* in horizontal cells. Image was created in Adobe Illustrator CS6. (**b**) Cycle-threshold (Ct) values of indicated mRNAs in PS, ONL, and InR samples. Shown are individual data points and means $\pm$ SD of n = 3. Statistics: one-way ANOVA with Holm-Sidak’s multiple comparisons test. *p $\le$ 0.05, ***p $\le$ 0.001.

The ReLayS method takes advantage of the structure of the retina, where photoreceptor inner and outer segments and cell bodies as well as inner retinal cells are organized in several distinct parallel layers (Fig. 1a). The method separates the PS, the ONL, and the InR from flat-mounted mouse half-retinas with the use of filter membranes (Fig. 1b). Detailed description of the ReLayS procedure is provided in the methods section. After separation of the retinal samples, proteins or RNA can be isolated and used in downstream applications including Western blotting (Figs. 2, 3 and 4), proteomics^14,^ or real-time PCR (Figs. 5 and 6). Protein yields from individual retinal layers corresponded to the yield from a whole half-retina (Table 1), indicating no considerable loss of material throughout the ReLayS procedure. Protein and RNA amounts isolated from PS, ONL, and InR samples of a single half-retina are sufficient for multiple assays (Table 1).

### Protein distribution in photoreceptor segments, outer nuclear layer, and inner retina

To validate the separation technique, we analyzed the levels of eight proteins with known retinal expression profiles in the ReLayS samples obtained from single half-retinas by Western blotting (Fig. 2). Phosphodiesterase 6 alpha (PDE6$\upalpha$) is the catalytic subunit of rod PDE6, which is essential for phototransduction and is located in the rod outer segments^15^. The exclusive appearance of PDE6$\upalpha$ in the PS samples (Figs. 2b,c and S1) indicated low contamination of the ONL and InR ReLayS samples with PS material. KH type-splicing regulatory protein (KHSRP), a multifunctional RNA binding protein involved in both transcriptional and posttranscriptional gene regulation^16^, was detected in the ONL and InR samples, both containing cell nuclei, but not in the PS samples (Fig. 2b,c). Thus, the PS samples were not, or not strongly, contaminated with material from the ONL. The photoreceptor marker proteins pyruvate kinase isomerase M2 (PKM2)^17^ and S-arrestin (SAG)^18^ were mainly present in the PS and ONL samples, indicating that indeed photoreceptor soma were mainly located in the ONL samples. Further, the citric acid cycle protein citrate synthase (CS) was predominantly found in PS and InR samples (Fig. 2b,c), suggesting that these samples had a high content of mitochondria. Since photoreceptor mitochondria are mainly located in the inner segments of photoreceptors, this indicates that the PS samples contain both, the outer and inner photoreceptor segments. The strong presence of the synaptic vesicle protein synaptophysin (SYP)^19^ in the ONL (Fig. 2b,c) indicated that the synaptic terminals of photoreceptor cells remained largely with the cell soma. Since the InR samples contain the IPL with the second synaptic complexes, the presence of SYP in these samples was expected (Fig. 2b,c). Protein kinase C alpha (PKC$\upalpha$) is a marker for retinal bipolar cells^20^ and was detected mainly in the InR but also in the ONL samples, indicating that some INL material was isolated together with the ONL. The ganglion cell-specific transcription factor BRN3A^21^ was detected exclusively in the InR samples, indicating low contamination of the PS and ONL samples with ganglion cell material.

In summary, PS samples contained both outer and inner segments with little contamination from the ONL. The ONL sample contained photoreceptor soma including the synaptic terminal and only little contamination from the PS layer. Separation between ONL and InR was less strict as the INL marker PKC$\upalpha$ was also found in the ONL samples and the photoreceptor markers PKM2 and SAG in the InR. Nevertheless, the method separated PS from the ONL with high accuracy, allowing the investigation of protein localization in these photoreceptor compartments. This was specifically tested by analysing the light-driven translocation of transducin in photoreceptors. Transducin is a heterotrimeric guanine nucleotide-binding protein that facilitates the activation of cGMP phosphodiesterase in the phototransduction cascade. In light conditions, transducin undergoes subcellular translocation from the outer segments to the inner segments and cell soma^22^. Thus, we tested PS and ONL samples obtained from dark- and light-adapted mice for the presence of the $\upalpha$-subunit of rod transducin (GNAT1) (Figs. 3 and S2). In dark-adapted mice, GNAT1 was detected exclusively in the PS samples, similar to the PDE6$\upalpha$ control. After light adaptation, however, GNAT1 but not PDE6$\upalpha$ was also massively present in the ONL samples, indicating that the light-induced translocation of GNAT1 is reliably detectable in the layers separated by our method.

We next analysed photoreceptor degeneration and reactive gliosis in the retina of a retinitis pigmentosa mouse model^23^ (Figs. 4 and S3). The heterozygous Rho-P23H knock-in mice (Rho^P23H/+^) carry one of the most frequent mutations in rhodopsin that causes autosomal dominant retinitis pigmentosa in humans^24^. As expected, reduced levels of rhodopsin (RHO) were detected in the PS samples of Rho^P23H/+^ mice at 3 months of age (Fig. 4a) due to ongoing photoreceptor degeneration. Additionally, increased levels of the signal transducer and activator of transcription 3 (STAT3) were detected in the PS and ONL samples of these mice (Fig. 4a). Increased STAT3 expression has been demonstrated in photoreceptors of light-damaged retinas and inherited photoreceptor degeneration before^25–27^.

Furthermore, we observed increased levels of the glial fibrillary acidic protein (GFAP) in the InR samples of Rho^P23H/+^ mice (Fig. 4b) indicating reactive gliosis in those retinas. Using this model of retinitis pigmentosa, we showed that the ReLayS method for preparation of PS, ONL, and InR samples can reliably be applied also on degenerative retinas. Since the technique also allows an unbiased screen of proteins by proteomics^14^, the intracellular location of proteins in various physiological and pathophysiological paradigms can be investigated.

Together, we found that the preparation of PS, ONL, and InR samples with the ReLayS method is highly reproducible and with low variability between replicates. The region of the outer limiting membrane, where the apical processes of Müller glial cells mark the transition of the inner segment to the photoreceptor cell body, and the OPL proved to be the “breaking points” for the successive retinal layer separation (Fig. 1).

### Localization of mRNA in photoreceptor segments, outer nuclear layer, and inner retina

To assess gene expression levels in different layers of the retina, RNA was extracted from PS, ONL, and InR samples obtained from single half-retinas. For RNA quality control, microcapillary electrophoretic RNA separation was performed and RNA integrity number (RIN) was calculated^28^ (Figs. 5a and S4). RIN ranged between 7.0 and 8.0 for PS, ONL, and InR samples obtained with the ReLayS method, indicating low levels of RNA degradation.

Since the samples originate from different cellular compartments and different cell types, it is extremely difficult, if not impossible, to determine reliable housekeeping genes for quantification of relative expression levels between the three different ReLayS fractions. This was exemplified by the Ct values for actin-beta (*Actb*) that variate considerably between the PS, ONL and InR samples (Table 2), making *Actb* not suitable as a reference housekeeping gene. Therefore, real-time PCR experiments were always performed with 10 ng of cDNA template and Ct values were plotted instead of relative expression values. Ideally, however, expression levels should be determined by absolute quantification methods such as digital PCR.

As for the proteins, we analysed the expression levels of six genes with known profiles within the retina^29^ (Fig. 5b,c). Expression of the rod photoreceptor-specific gene rhodopsin (*Rho*) was detected in the PS and ONL, but to lower levels also in the InR samples. Aquaporin-4 (*Aqp4*) mRNA, a water-specific channel expressed in Müller glia cells^30^, was detected in the ONL and InR samples (Fig. 5c), similar to *Slc2a3* encoding the glucose transporter GLUT-3, which is expressed by inner retinal cells^31^. The amacrine cell-specific gene myogenic factor 6 *Myf6* was also detected predominantly in the InR samples and to lower levels in the ONL (Fig. 5c). Finally, expression of the smooth muscle cell-specific actin *Acta2* and the ganglion cell-specific transcription factor *Pou4f1*^21^ was highly enriched in the InR samples containing the retinal vessels and the ganglion cells. These data indicate a good separation of the layers by ReLayS but with some contamination of the ONL with genes expressed in the INL but not GCL. The contamination of the ONL with cells from the INL was further indicated by additional expression data for bipolar (*Vsx2* and *Cabp5*) and horizontal cell (*Marc1*) marker genes (Fig. 6). Thus, data from ONL and InR samples have to be interpreted with caution and should be verified by additional methods such as in situ hybridization. Nevertheless, we showed that ReLayS samples can be used—with some limitations—also for gene expression analysis by semi-quantitative RT-PCR.

**Table 1:** Protein and RNA yields from PS, ONL, and InR samples obtained from flat-mounted half-retina (R) prepared with the ReLayS method.

	PS	ONL	InR	R
protein concentration [ng/$\upmu$l] mean ± SD	481 ± 73.3	317 ± 48.2	470 ± 63.4	1051 ± 170
total protein yield [$\upmu$g] mean ± SD	45.7 ± 7	30.1 ± 4.6	44.6 ± 6	123.2 ± 16.8
RNA concentration [ng/$\upmu$l] mean ± SD	21.4 ± 0.7	7 ± 2.7	15.3 ± 0.9	–
total RNA yield [ng] mean ± SD	342 ± 10.9	113 ± 43.4	244 ± 14.5	–

**Table 2:** Real-time PCR for *Actb* in PS, ONL, and InR.

	PS	ONL	InR
Ct value mean ± SD	20.8 ± 0.49	23 ± 1.49	19.6 ± 0.52

## Discussion

Understanding the physiology of the retina and especially of the highly polar photoreceptors is essential not only for describing the mechanisms required for normal vision but also for the development of successful therapies to prevent or slow retinal degenerative diseases. However, the molecular analysis of photoreceptors is a challenge due to the heterogeneity of the retinal tissue and the lack of easy and reliable methods for cell separation. In this study, we introduce the ReLayS methoda simple method for the separation of photoreceptor inner and outer segments from the outer nuclear layer, and the inner retina. The layer-specific material isolated from half-retinas was sufficient for protein isolation and Western blotting or RNA isolation and real-time PCR studies. It was also of sufficient quality and quantity to perform layer-specific proteomics^14^.

The reproducibility of the ReLayS method was verified by Western blotting and real-time PCR for proteins and genes with known expression profiles in the retina (Figs. 2 and 5). The distribution of marker proteins showed that the material after the first isolation contained almost exclusively photoreceptor outer and inner segments without strong contamination by the ONL. The third isolate was mostly of inner retinal origin but also contained some material from the ONL as indicated by the presence of PKM2 and SAG in the InR samples. PS, however, were completely absent in this isolate. Similarly, the ONL isolate was devoid of PS material but was somewhat contaminated by inner retinal cells. Rupture of the retinal cells due to the repeated freezing and thawing of the tissue during ReLayS could lead to some spill-over of intracellular material in the ONL and InR samples. In summary, the first isolate contained almost exclusively photoreceptor segments, the second was highly enriched in ONL material and the third in cells of the inner retina. ReLayS is thus especially useful to determine whether a protein localizes to the segments or the soma of photoreceptors and whether this localization is affected by environmental (e.g., light) or genetic (mutations) factors, or by degenerative processes.

It is also possible to investigate the localization of specific RNAs by real-time PCR. However, such studies need to be interpreted with caution, as the high sensitivity of the method allows detection of even small amounts of contamination from other layers. In addition, determining differences among the three isolates in the expression level of a particular gene may only be possible by quantitative but not semi-quantitative real-time PCR as the genes required for normalization may also be present at different levels in the three isolates (Table 2). Nevertheless, analysing the presence of a particular RNA in the three isolates may help to address the expression pattern in various physiological and pathophysiological situations.

ReLayS is inspired by the method developed by Rose et al.^13^ but has the advantage that it not only isolates the photoreceptor inner and outer segments together but also generates samples highly enriched for material from the ONL or the inner retina. It is a simple and useful tool to address specific research questions including the distribution of proteins and possibly metabolites in various situations including development, ageing, and degenerative diseases. Combining ReLayS with simple Western blotting or more powerful downstream applications such as proteomics or metabolomics may help to advance our understanding of basic physiological and degenerative processes in the retina.

## Methods

### Mice

All animal experiments adhered to the standards of the ARVO Statement for the Use of Animals in Ophthalmic and Vision Research and the ARRIVE guidelines. The experimental protocols were approved by the Veterinary Office of Canton Zurich, Switzerland, under the animal experimentation licence ZH091/2019. 129S6 wild-type mice (Taconic) and heterozygous Rho^P23H/+^ knock-in mice (Jackson Laboratory) were maintained in the Laboratory Animal Services Center facilities of the University of Zürich, with a 14h:10h light:dark cycle and access to food and water ad libitum. Euthanasia was performed with CO$_{2}$ exposure followed by decapitation. All samples were collected between 1 and 2 pm to prevent a potential influence of the circadian rhythm or time since light onset.

### Dark- and light-adaptation of mice

For testing transducin migration in photoreceptors, all mice were dark-adapted overnight. Dark-adapted mice were kept in darkness until euthanasia. ReLayS was performed under dim red light in these mice. To induce translocation of GNAT1, mice were exposed to 1500 lux for 1 h and ReLayS was carried out immediately thereafter under normal room light.

### ReLayS–retinal layer separation

Retinas were rapidly dissected through a slit in the cornea and placed in ice-cold PBS. Remaining vitreous was carefully removed using a pair of forceps and retinas were halved through the optic nerve head. For easier flattening of the tissue, a small incision was made in the peripheral retina, opposite to the optic nerve. Using a pair of forceps, half-retinas were gently placed on a microscope slide immersed in the PBS so that the ganglion cell layer was facing up. The glass slide with the tissue was then gently lifted out of the PBS, causing flattening of the retina. A small piece of Durapore filter membrane (Merck Millipore; DVPP04700) was placed on top of the half-retina and few drops of PBS were applied, causing the adherence of the ganglion cell layer to the membrane. The Durapore membrane was lifted from the glass slide and placed on a paper towel to drain the liquid, further increasing the adhesion of the retina to the membrane. The draining procedure was repeated 3 times by placing the Durapore membrane on a dry paper towel and adding a drop of PBS on top of the retina. The half-retinal flat mounts were frozen on a metal platform cooled with dry ice and stored in Eppendorf tubes at $-$80 $^{\circ }$C until further use.

For separation of PS and ONL, the membrane with the frozen retinal flat mount was placed on top of a Whatman filter paper (3MMChr; GE Healthcare; 3030-917), which was slightly moistened (just enough to cause a moisture-induced colour changeover of the Whatman paper, visible under dissection microscope) with PBS at room temperature. After the tissue was completely thawed, a small piece of dry Durapore filter membrane was placed carefully on top of the retina and left in place for 10 seconds to allow the PS to adhere to it. After carefully lifting the top membrane from the tissue, the PS remained adhered to it, and thus separated from the flat-mounted retina. The PS-containing membrane was frozen on a metal platform cooled with dry ice, placed in Eppendorf tubes, and stored frozen until further use. The remaining flat-mounted tissue was re-frozen on a metal platform cooled with dry ice and the separation procedure was repeated to separate the ONL from the InR. Finally, the InR was transferred to a new Durapore membrane by repeating the same procedure but moistening the Whatman filter paper till liquid drops were visible on its surface. This is necessary in order to separate the InR sample from the layer-contaminated rim of the tissue. All Durapore membranes were trimmed to the minimum size around the tissue, frozen as outlined above, and stored at $-$80 $^{\circ }$C until further processing.

### Protein isolation and western blot

For isolating proteins from the separated retinal layers or flat-mounted half-retinas, 81 $\upmu \hbox {l}$ ice-cold Tris-HCl (100 mM, pH 8.0) and 9 $\upmu \hbox {l}$ protease inhibitors (Sigma-Aldrich; P2714) were added to the tubes containing the membranes with the PS, ONL, or InR. Tissues were homogenized by sonication (13 pulses of 0.3 seconds and 30% amplitude) with an ultrasonic homogenizer (Branson 450 Digital Sonifier). 10 $\upmu \hbox {l}$ of 10% sodium dodecyl sulfate (SDS in Tris-HCl, pH 8.0) was added and samples were incubated for 10 minutes at 75 $^{\circ }$C with shaking at 300 rpm. Samples were centrifuged at 15.2 g for 1 min in a conventional benchtop microcentrifuge at RT and the supernatants were transferred to a new Eppendorf tube. Benzonase (4.5 units/100 $\upmu$l; MilliporeSigma; E1014) was added and samples incubated at 37 $^{\circ }$C for 1 h without shaking to digest DNA/RNA present in the isolates. Protein concentrations were measured with the Pierce BCA Protein Assay Kit (Thermo Scientific; 23225) using a plate reader (BioTek, Synergy HT) according to the manufacturer’s instructions. Protein samples were stored at $-$20 $^{\circ }$C until further use.

Western blotting was performed to detect proteins of interest in the samples. Briefly, 5 $\upmu$g of proteins were run on a 10% SDS PAGE and blotted onto a nitrocellulose membrane (BioRad, 1620112) using the Trans-Blot Turbo Transfer System (BioRad, 1704150). The membrane was blocked in 5 % nonfat dry milk (Bio-Rad Laboratories; Blotting-Grade Blocker 1706404) in TBST (Tris-Buffered Saline with 0.1% Tween) for 1 h at RT and incubated with the following primary antibodies overnight at 4 $^{\circ }$C: rabbit anti-PDE6$\upalpha$ (1:750; Abcam, ab5659), rabbit anti-KHSRP (1:5000; Novus Biologicals, NBP1-18910), rabbit anti-PKC$\upalpha$ (1:1000; Sigma-Aldrich, P4334), rabbit anti-PKM2 (1:1000; Cell Signaling, 3198S), rabbit anti-CS (1:1000; GeneTex, GTX110624), mouse anti-SYP (1:1000; Novocastra, NCL-L-SYNAP-299), rabbit anti-SAG (1:500; Affinity BioReagents, PA1-731), mouse anti-BRN3A (1:500; Chemicon, MAB1585), rabbit anti-GNAT1 (1:200; Santa Cruz Biotechnology, sc-389), mouse anti-RHO (1:8000; Sigma-Aldrich, O4886), rabbit anti-STAT3 (1:500; Cell Signaling, D3Z2G), and mouse anti-GFAP (1:1000; Sigma-Aldrich, G3893). Following three washing steps with TBST, membranes were incubated with respective secondary antibodies (donkey anti-rabbit IgG HRP or goat anti-mouse IgG HRP; 1:10000) for 1 h at RT. Membranes were washed with TBST and secondary antibodies were detected by chemiluminescence (PerkinElmer) using X-ray films (Fujifilm, 47410 19236). Densitometry was performed with ImageJ (National Institutes of Health) and data were visualized with GraphPad Prism (GraphPad Software Inc.) software.

### RNA extraction, cDNA synthesis, and semi-quantitative real-time PCR

Total RNA was isolated from the tissue on the membrane with an RNA isolation kit (Thermo Fisher PicoPure RNA Isolation Kit, KIT0204) including an on-column DNaseI treatment. RNA electrophoresis was performed with the Agilent 2200 TapeStation (Agilent Technologies) using the standard RNA ScreenTape Assay for the PS and InR samples and the High Sensitivity RNA ScreenTape Assay for the ONL samples. Results were analysed with the TapeStation Analysis Software 3.2. cDNA synthesis was carried out with oligo-(d)T primers and M-MLV reverse transcriptase (Promega). For semi-quantitative real-time PCR, 10 ng cDNA was amplified using the PowerUp SYBR Green Master Mix (Thermo Fisher Scientific) and specific primer pairs (Table 3) in the ABI QuantStudio 3 system (Thermo Fisher Scientific). Primers were designed, whenever possible, to span large intron sequences or exon-exon boundaries to avoid genomic DNA amplification and tested for specificity and efficiency before experiments. Actin-beta (*Actb*) was not suitable as a reference housekeeping gene due to variable expression levels in the three different ReLayS samples (Table 2).

**Table 3:** Primers used for real-time PCR.

Gene name	Forward (5’-3’)	Reverse (5’-3’)
*Rho*	CTTCACCTGGATCATGGCGTT	TTCGTTGTTGACCTCAGGCTTG
*Aqp4*	TACTGGAGCCAGCATGAATC	CCACATCAGGACAGAAGACA
*Slc2a3*	AGGTCACCCAACTACGTCCA	CACCCGCGTCCTTGAAGATT
*Acta2*	CCCTGAAGAGCATCCGACAC	ACAGCACAGCCTGAATAGCC
*Myf6*	GTGGACCCCTACAGCTACAA	CTCCTCCTTCCTTAGCAGTTA
*Pou4f1*	CGCCGCTGCAGAGCAACCTCTT	TGGTACGTGGCGTCCGGCTT
*Actb*	CAACGGCTCCGGCATGTGC	CTCTTGCTCTGGGCCTCG
*Vsx2*	CTTCCCGGCTTCTACACACA	TCGGTCACTGGAGGAAACATC
*Cabp5*	CAATGCAGTTTCCAATGGGTC	CCAACTCAGTCAACTCCATCT
*Marc1*	TCCTCCAGTGCAGAGTGCAT	AGGCGACAGGACTGCATCTT

### Statistical analysis

Statistical analysis of the Western blot densitometry data and real-time PCR data was performed using Student’s *t*-test or one-way ANOVA with Holm-Sidak’s multiple comparisons test as indicated in figure legends with GraphPad Prism (GraphPad Software Inc.) software. *P*-values $< 0.05$ were considered to show significant differences.

## Supplementary Information

Supplementary Information.

## Data Availability

All data generated or analysed during this study are included in this published article.
