# Supplementary material for: Retinal Layer Separation (ReLayS) method enables the molecular analysis of photoreceptor segments and cell bodies, as well as the inner retina
Source: Sci Rep. 2022 Nov 23;12:20195. doi: 10.1038/s41598-022-24586-8 (PMC9691741; doi:10.1038/s41598-022-24586-8)
Supplement: Supplementary file 1 — Supplementary Information. [file 41598_2022_24586_MOESM1_ESM.pdf]

Supplementary Information

Retinal layer separation (ReLayS) method enables the molecular analysis of photoreceptor segments and cell bodies, as well as the inner retina

Vyara Todorova[1], Luca Merolla[1], Duygu Karademir[1], Gabriele M. Wögenstein[1], Julian Behr[1], Lynn J. A. Ebner[1], Marijana Samardzija[1], and Christian Grimm[1,*]

[1]Laboratory for Retinal Cell Biology, Department of Ophthalmology,University Hospital Zurich, University of Zurich, Schlieren, 8952, Zurich, Switzerland

[*]cgrimm@opht.uzh.ch

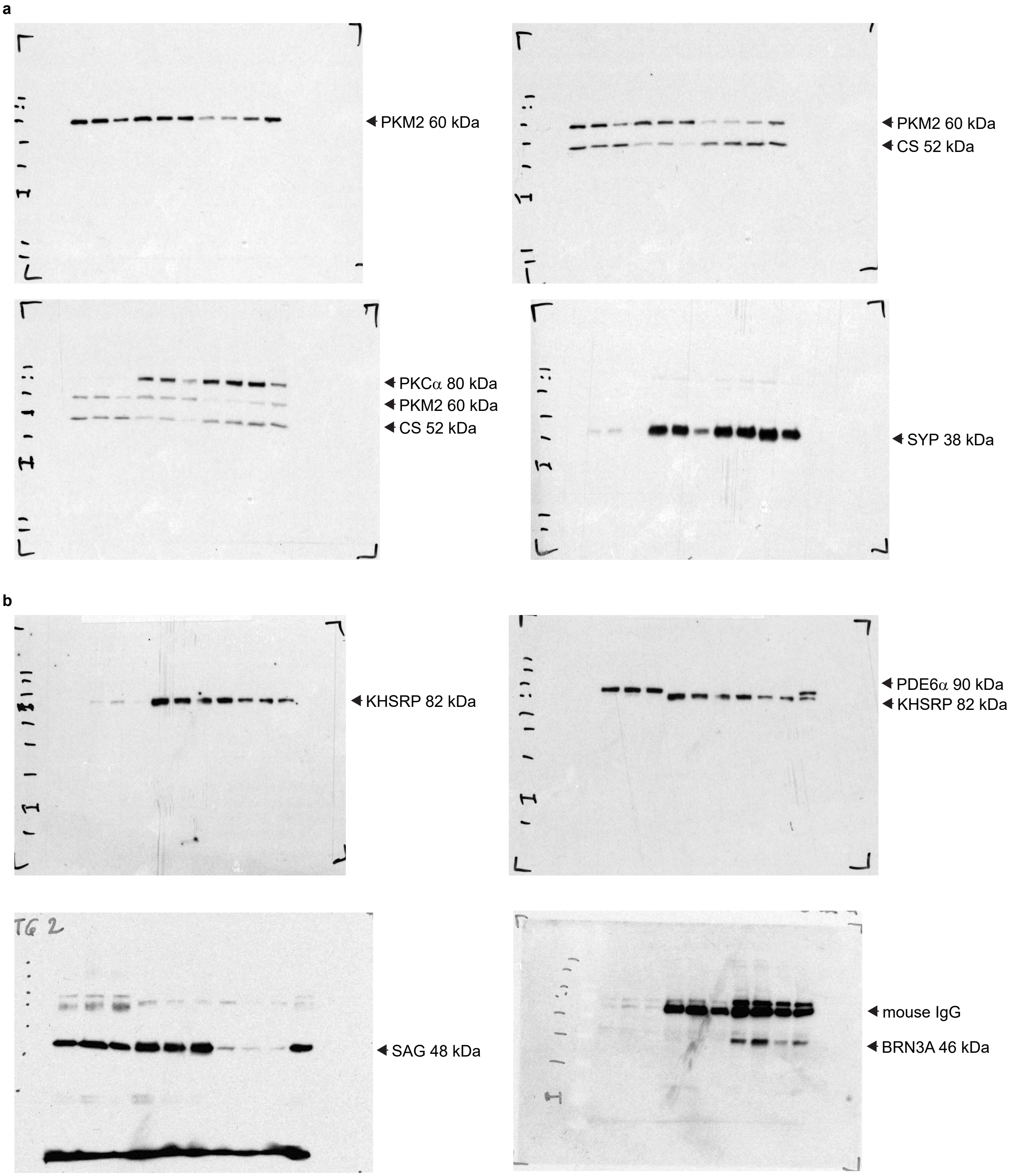

Figure S1. Related to Figure 2b. (**a**) Detection of PKM2, CS, PKCα, and SYP in PS, ONL, InR, and whole retina by Western blotting. (**b**) Detection of KHSRP, PDE6α, and BRN3A in PS, ONL, InR, and whole retina by Western blotting.

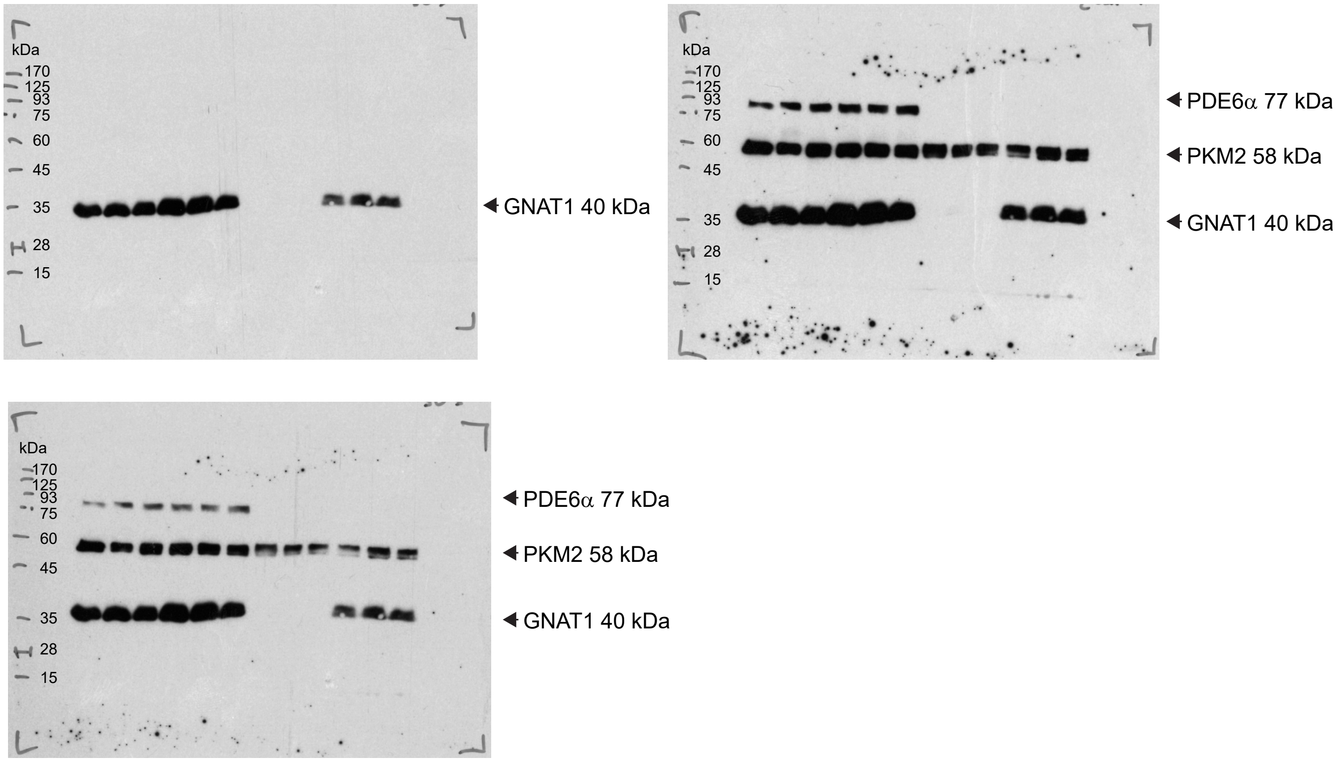

Figure S2. Related to Figure 3. Detection of GNAT1, PDE6α, and PKM2 in PS and ONL by Western blotting.

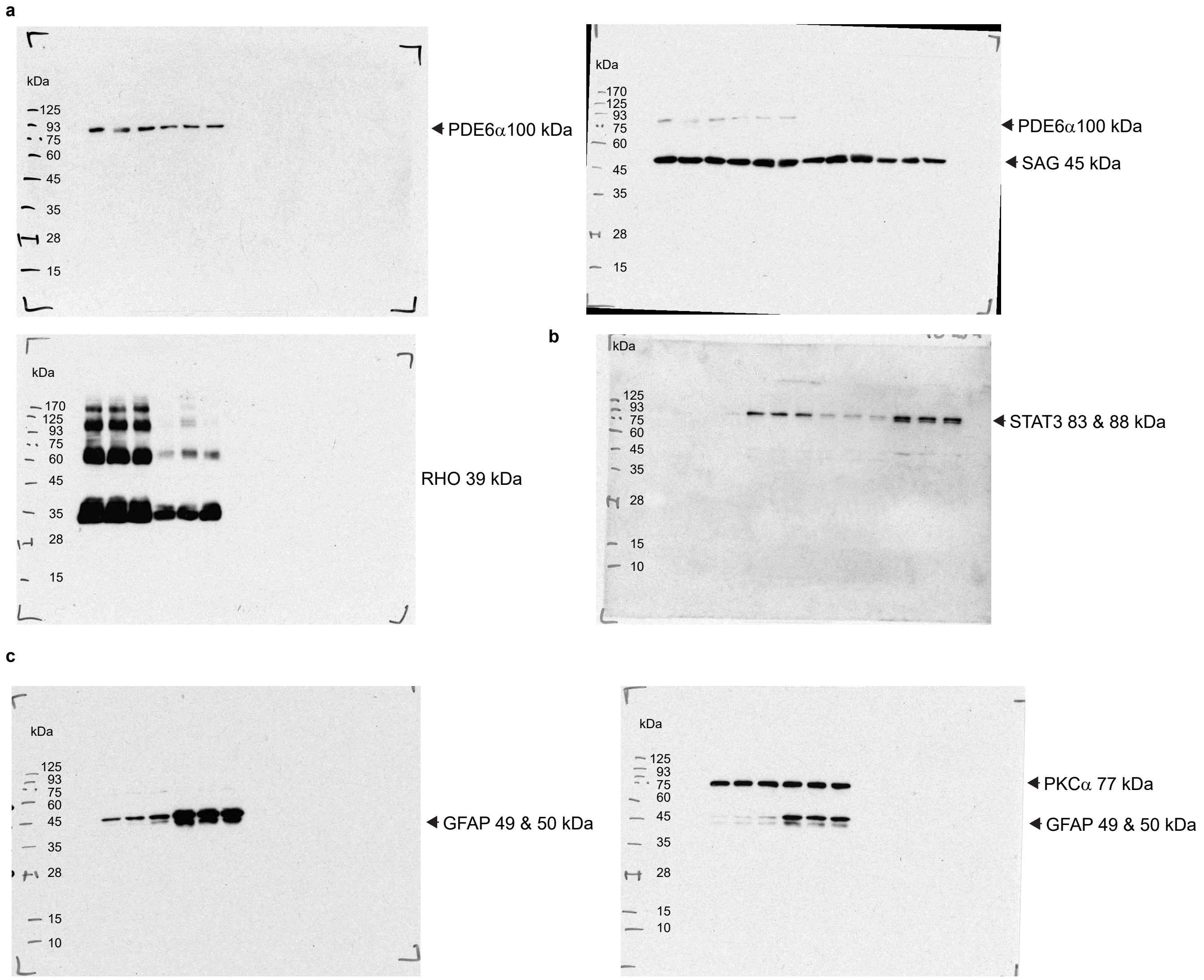

Figure S3. Related to Figure 4. (**a**) Detection of PDE6α, SAG, and RHO in PS and ONL by Western blotting. (**b**) Detection of STAT3 in PS and ONL by Western blotting. (**c**) Detection of GFAP and PKCα in InR by Western blotting.

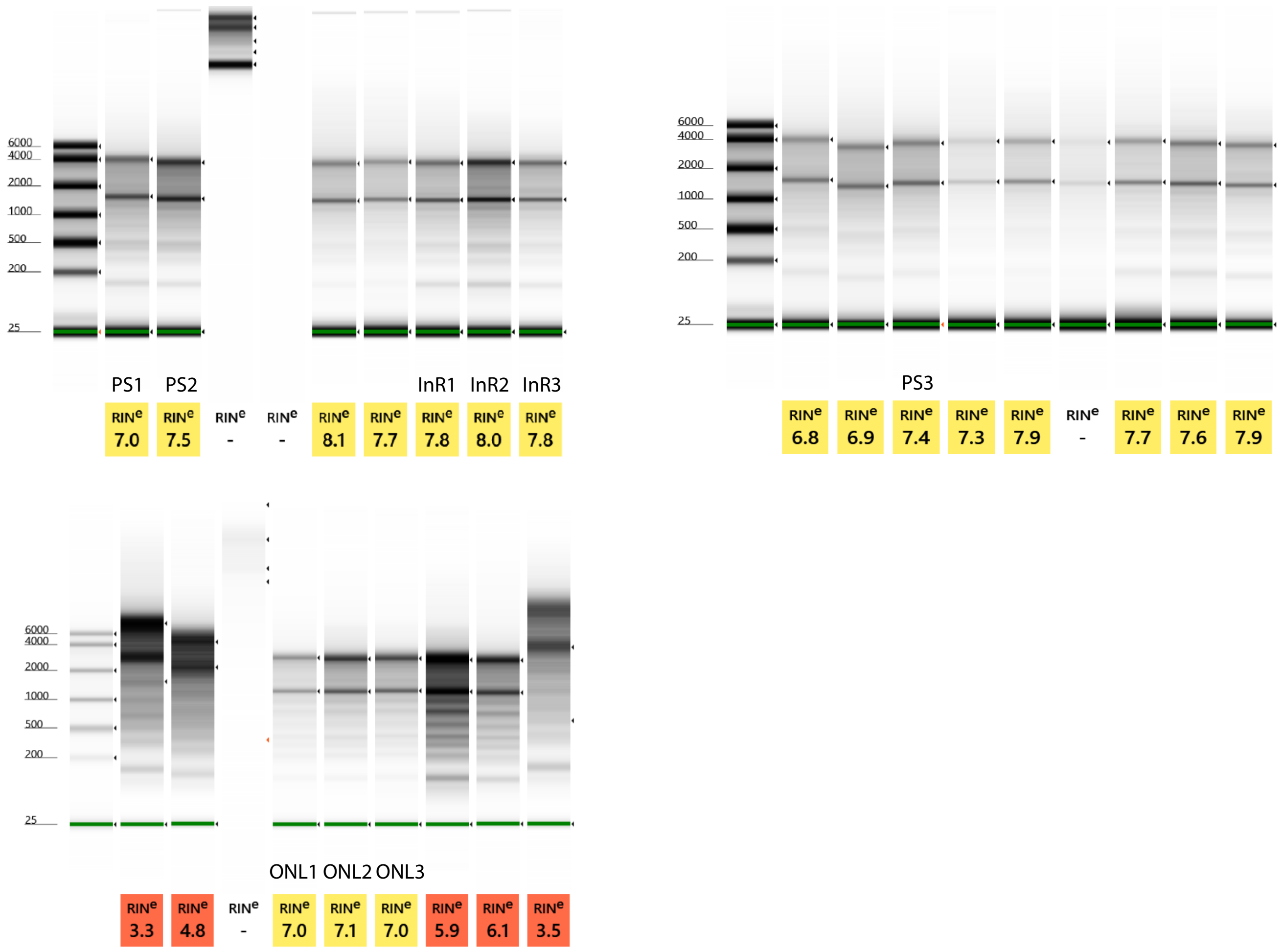

Figure S4. Related to Figure 5a.
